# CA125 and Ovarian Cancer: A Comprehensive Review

**DOI:** 10.3390/cancers12123730

**Published:** 2020-12-11

**Authors:** Parsa Charkhchi, Cezary Cybulski, Jacek Gronwald, Fabian Oliver Wong, Steven A. Narod, Mohammad R. Akbari

**Affiliations:** 1 Women’s College Research Institute, University of Toronto, Toronto, ON M5S 1B2, Canada; parsa.charkhchi@mail.utoronto.ca (P.C.); fabian.wong@mail.utoronto.ca (F.O.W.); steven.narod@wchospital.ca (S.A.N.); 2 International Hereditary Cancer Center, Department of Genetics and Pathology, Pomeranian Medical University, 71-252 Szczecin, Poland; cezarycy@pum.edu.pl (C.C.); jgron@pum.edu.pl (J.G.); 3 Institute of Medical Science, Faculty of Medicine, University of Toronto, Toronto, ON M5S 1A8, Canada; 4 Dalla Lana School of Public Health, University of Toronto, Toronto, ON M5T 3M7, Canada

**Keywords:** ovarian cancer, CA125, screening, point-of-care

## Abstract

**Simple Summary:**

CA125 has been the most promising biomarker for screening ovarian cancer; however, it still does not have an acceptable accuracy in population-based screening for ovarian cancer. In this review article, we have discussed the role of CA125 in diagnosis, evaluating response to treatment and prognosis of ovarian cancer and provided some suggestions in improving the clinical utility of this biomarker in the early diagnosis of aggressive ovarian cancers. These include using CA125 to screen individuals with symptoms who seek medical care rather than screening the general population, increasing the cutoff point for the CA125 level in the plasma and performing the test at point-of-care rather than laboratory testing. By these strategies, we would detect more aggressive ovarian cancer patients in stages that the tumour can be completely removed by surgery, which is the most important factor in redusing recurrence rate and improving the survival of the patients with ovarian cancer.

**Abstract:**

Ovarian cancer is the second most lethal gynecological malignancy. The tumour biomarker CA125 has been used as the primary ovarian cancer marker for the past four decades. The focus on diagnosing ovarian cancer in stages I and II using CA125 as a diagnostic biomarker has not improved patients’ survival. Therefore, screening average-risk asymptomatic women with CA125 is not recommended by any professional society. The dualistic model of ovarian cancer carcinogenesis suggests that type II tumours are responsible for the majority of ovarian cancer mortality. However, type II tumours are rarely diagnosed in stages I and II. The recent shift of focus to the diagnosis of low volume type II ovarian cancer in its early stages of evolution provides a new and valuable target for screening. Type II ovarian cancers are usually diagnosed in advanced stages and have significantly higher CA125 levels than type I tumours. The detection of low volume type II carcinomas in stage IIIa/b is associated with a higher likelihood for optimal cytoreduction, the most robust prognostic indicator for ovarian cancer patients. The diagnosis of type II ovarian cancer in the early substages of stage III with CA125 may be possible using a higher cutoff point rather than the traditionally used 35 U/mL through the use of point-of-care CA125 assays in primary care facilities. Rapid point-of-care testing also has the potential for effective longitudinal screening and quick monitoring of ovarian cancer patients during and after treatment. This review covers the role of CA125 in the diagnosis and management of ovarian cancer and explores novel and more effective screening strategies with CA125.

## 1. Introduction

In 2018, 295,000 new ovarian cancer cases were diagnosed, leading to the death of more than 180,000 women worldwide [1]. The 5-year survival rate for early-stage (I, II) ovarian cancer is about 90%, whereas only 20–40% of late-stage (III, IV) ovarian cancer patients live beyond five years [2,3]. Despite the advances in treatment, ovarian cancer patients have an overall 5-year survival rate of 45%, making this disease the second most lethal gynecological malignancy [4]. About three-quarters of invasive epithelial ovarian cancers show symptoms only at an advanced stage, whereas early-stage patients present with only a few mild and unspecific symptoms [5]. An estimated 22% of patients with high-grade serous carcinomas (HGSC) have germline mutations in BRCA1/2 genes [6]. Carriers of BRCA1 and BRCA2 mutations have a lifetime risk of 40–60% and 11–30%, respectively [7]. The risk for ovarian cancer is inversely correlated with parity, oral contraceptive usage, hysterectomy, and tubal ligation surgery. A family history of ovarian cancer is known as the most important risk factor. Other modest risk factors include obesity and hormone replacement therapy [8,9,10].

In general, ovarian cancer comprises a heterogeneous group of malignant tumours affecting multiple locations within the peritoneal cavity [11,12]. Efforts for the development of effective screening methods are yet to provide a sufficient survival advantage. Therefore, routine screening in asymptomatic average-risk women is not recommended by any professional society [13]. The dualistic ovarian carcinogenesis model condenses the major histopathological subtypes into type I and type II based on clinical, genetic, and developmental components. In terms of diagnosis, about 30% of diagnosed ovarian carcinomas are type I, and 70% are type II [14,15]. Type I tumours are usually confined to the ovaries (stage I) and have a favourable prognosis, accounting for 10% of ovarian cancer-related deaths. On the contrary, the more aggressive type II tumours are diagnosed in advanced stages (III, IV), where a cure is not likely [16,17]. Type II tumours are responsible for 90% of ovarian cancer-related deaths. As a result, some groups have proposed that type II tumours should become the focus of extensive screening to observe significant improvements in outcome [17]. The development of new and more effective screening strategies that target type II tumours has the potential to detect aggressive cancers in a stage where optimal treatment is possible.

Tumour biomarkers have played an essential role in the detection and management of ovarian cancer. Numerous ovarian cancer biomarkers have been the subject of extensive and intensive studies. Among these biomarkers, Cancer Antigen 125 (CA125), also referred to as Carbohydrate Antigen 125, has played the most significant role in screening, detecting, and managing ovarian cancer for the last four decades. CA125 is a high molecular weight mucinous glycoprotein found on the surface of ovarian cancer cells. This antigen is then shed and quantified in serum samples of ovarian cancer patients. Serum CA125 levels are elevated in 50% of early-stage tumours, which are mostly type I ovarian cancers and 92% of advanced-stage tumours, which are mostly type II ovarian cancers [18,19]. However, due to the low incidence of ovarian cancer, screening average-risk women with CA125 results in a considerable number of false positives [20]. This review aims to cover the role of CA125 in the diagnosis and management of ovarian cancer and reviews the recent literature on novel screening techniques involving CA125.

## 2. Prognosis and Survival in Ovarian Cancer

Less than 20% of advanced-stage ovarian cancer patients survive beyond ten years [21]. Surgery and chemotherapy have been used as the main ovarian cancer treatments for many decades. Primary debulking surgery is the preferred initial treatment option for patients with advanced-stage ovarian cancer [22]. Complete resection of tumours leading to no visible residual disease during primary cytoreductive surgery is one of the major prognostic factors for ovarian cancer patients [23]. Advanced stage ovarian cancer patients (stages IIIc and IV) who have undergone optimal primary debulking surgery (no visible residual disease) have a better seven-year survival than women with any residual disease remaining after surgery (73.6% vs 21%; *p* < 0.0001) [24]. Stage IIIa/b patients are also more likely to achieve optimal resection than stage IIIc/d [25,26]. Optimal primary debulking surgery followed by chemotherapy reduces the number of residual cancer cells, leading to survival improvements. Tumour respectability is dependent on multiple factors, including the location, size, mutation status, and surgical complexity [27]. The increased survival for optimally resected tumours is independent of factors such as stage at diagnosis, mutation status, or surgical complexity [27,28,29]. Median survival for patients with recurrent ovarian cancer is 12–24 months, while the disease is considered incurable [23,30]. Patients without intra-abdominal recurrence rarely die from ovarian cancer. The chance of recurrence is much higher in patients with any residual disease remaining after primary debulking surgery than those with no residual disease after surgery [29]. 

Different subtypes of ovarian cancer have variable stage distributions. Type I and II ovarian cancers are different diseases with varying developmental and prognostic implications (Table 1). Type I tumours develop in a stepwise manner from borderline tumours and are usually diagnosed in earlier stages (I, II). Precursors of type I tumours are relatively easy to discover, whereas precursors of type II tumours are more challenging to detect. The majority of type II tumours are known to develop de novo from the fallopian tubes in the form of microscopic serous tubal intraepithelial carcinomas (STIC). Such tumours are usually diagnosed in advanced stages (III, IV) and have distinct morphologic and molecular genetic features compared to type I tumours. Early diagnosis of ovarian cancer in stage I was thought to be the most important prognostic factor for ovarian cancer patients. However, a large proportion of aggressive HGSCs (type II tumours) are discovered when they have already invaded the peritoneal cavity. The emphasis on early detection of ovarian cancer in stages I and II has limitations, including a limited effect on survival and quality of life due to the diagnosis of more “good prognosis” type I tumours [14,31,32,33]. The lack of improvement in survival has led to a “crisis of confidence” in the field [34]. Therefore, the lower mortality associated with early-stage ovarian cancer appears to be due to the biological and developmental differences between type I and II tumours rather than the stage distribution at diagnosis [34]. Consequently, multiple groups have suggested that the goal of screening should be the diagnosis of low volume type II tumours in a stage where a status of no residual disease is more likely to be achieved through cytoreductive surgery [16,34]. 

## 3. Early Detection of Ovarian Cancer

Multiple serum tumour biomarkers, radiological imaging techniques, and risk stratification algorithms have been the focus of extensive research for the early diagnosis of ovarian cancer. Women with early-stage ovarian cancer experience few symptoms. On the contrary, the disease’s progression may be associated with more visible unspecific symptoms, including bloating, abdominal pain and swelling, early satiety, and frequent or urgent urination. Additionally, extreme weight loss and bowel obstruction may follow as the disease progresses to advanced stages [35]. The value of early detection of ovarian cancer, especially in the pre-symptomatic stages where the prognosis is much more favourable, cannot be overstated. However, tumours that are more likely to be detected in the early stages (type I tumours) are associated with better survival due to their molecular structure rather than their stage at diagnosis [17]. Therefore, ovarian cancer screening in an asymptomatic average-risk woman is currently not the most effective option [13]. 

### 3.1. CA125

Biomarkers identify circulating tumour elements, proteins overexpressed by tumours, or components of the immune response to the tumour in body fluids such as blood and urine [36]. Ovarian cancer protein biomarkers are currently used for monitoring disease progression. However, some ovarian cancer serum biomarkers are also used as screening strategies for high-risk populations. An ideal screening serum tumour marker has sufficient specificity and sensitivity to reach a positive predictive value (PPV) of 10% analogous to 1 cancer diagnosed out of 10 positive test results. There are two strategies for improving the PPV of screening tests. One approach is changing the screening population from asymptomatic women to symptomatic women who will have a higher frequency of ovarian cancer. The other approach is increasing the cutoff point for reducing false-positive results. Due to the low prevalence of ovarian cancer, the ideal screening test must have a sensitivity above 75% and a specificity of at least 99.6% [37,38,39,40]. The only strategy we have for adjusting the sensitivity and specificity of a test is changing the cutoff point. In general, tumour biomarkers become clinically significant upon an accurate prediction of the disease at screening, management, and follow up phases of treatment. Currently, no single ovarian cancer biomarker performs well in all three phases. 

The ovarian cancer biomarker CA125 has been investigated thoroughly regarding ovarian cancer screening, detection, and progression. Bast and colleagues first described CA125 in 1981 by developing a monoclonal antibody (OC125) against this antigen. In the original study, BALB/c mice were immunized with the OVCA-433 cell line isolated from the ascites fluid of a patient with ovarian serous papillary cystadenocarcinoma [41]. A radioimmunoassay was developed in 1983 to detect CA125 in serum using the 35 U/mL threshold. About 80% of patients with ovarian cancer exhibited elevated CA125 levels measured using this homologous double determinant assay, while only 1% of healthy women in the study had elevated CA125 levels beyond 35 U/mL. However, the authors mentioned that CA125 levels’ normalization should not be the only evidence for disease remission since small residual tumours can be present in women with normal CA125 levels [42]. The upper limit of 35 U/mL has not changed since the first report.

Further analysis revealed that CA125 is a large glycoprotein with variable weights due to variation in glycosylation [43]. The amino acid analysis showed that CA125 has a high serine, threonine, and proline content seen in mucin-like molecules [44]. Partial cDNA for the CA125 protein component was cloned in 2001 by Yin and Lloyd. Consequently, CA125 was found to be an epitope on a glycoprotein encoded by the MUC16 gene, located on the short arm of chromosome 19, at 19p13.3. Furthermore, MUC16 has multiple epitopes recognized by different antibodies [43]. Specifically, CA125 has two main antigenic domains, separately binding OC125-like antibodies (group A) and M11-like antibodies (group B) [19,45]. CA125II is another heterologous assay that replaced the original CA125 assay. CA125II uses both M11 on the solid phase and OC125 as a probe and has less intraassay variation [46,47]. 

Understanding the variation between subtypes of ovarian cancer is an important factor for the development of useful biomarkers. CA125 expression varies between different subtypes of ovarian cancer. HGSC and endometroid ovarian cancers have a higher expression of CA125 compared to other subtypes [48]. Generally, serous tumours have higher CA125 concentrations, and mucinous ovarian cancers have the lowest [42]. Likewise, type I and II tumours have different CA125 expression patterns (Table 2). Patients with type I ovarian cancer are usually younger and are more frequently diagnosed in the asymptomatic early stages. Similarly, multiple studies have shown significantly higher CA125 levels in type II patients than patients with type I tumours [15,49,50,51,52,53,54,55]. Type II tumours account for 75% of ovarian carcinomas and 90% of ovarian cancer mortality [16]. In a study by Lu et al. on 14 ovarian cancer serologic markers, CA125 showed the highest discriminatory power for type II tumours compared to healthy controls. Additionally, serum levels of CA125 showed more dramatic fluctuations in patients with type II tumours than type I patients [56]. The role of CA125 in predicting suboptimal cytoreduction was also investigated in a prospective multicenter trial involving stage III and IV patients undergoing primary cytoreductive surgery. Six criteria were associated with suboptimal cytoreduction (>1 cm residual cancer), including CA125 levels above 500 U/mL [57]. 

#### 3.1.1. CA125: Physiological Functions

**Figure 1:**
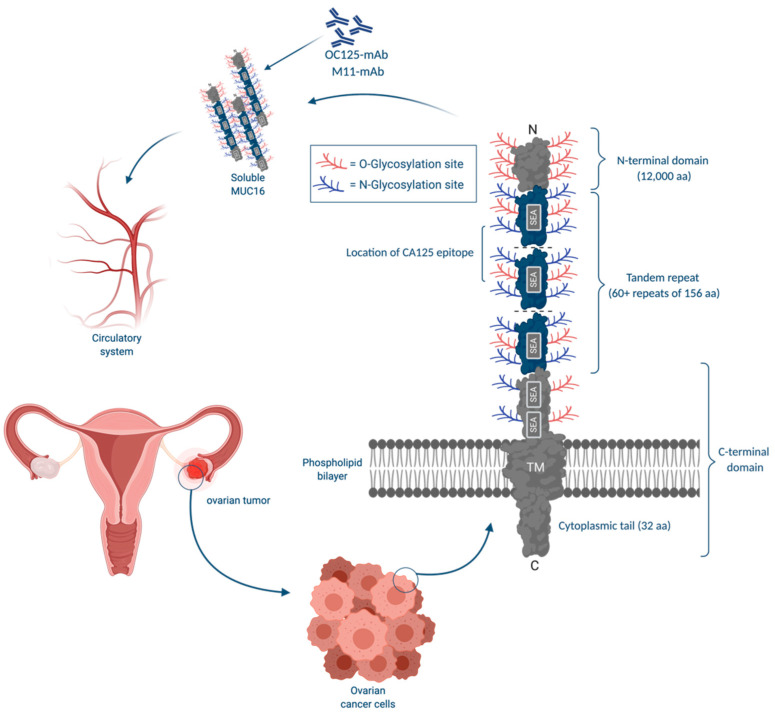
Illustration of MUC16(CA125) structure and its role in ovarian cancer. MUC16 has cytoplasmic, transmembrane, and extracellular components that harbour variable O- and N-glycosylation sites. The peptide component of MUC16 has approximately 22,152 amino acids. The N-terminal domain of MUC16 has 12,000 amino acids and exclusively harbours O-glycosylation. A considerable portion of its peptide component is composed of the tandem repeat region with 60+ repeats of 156 amino acids. MUC16 harbours about 56 sea-urchin, enterokinase, and agrin (SEA) domains. SEA domain is a shared feature among mucins, this domain is involved in cleavage and association of MUC16 subunits. The transmembrane domain is followed by a 32 amino acids cytoplasmic tail with possible phosphorylation sites. MUC16 is cleaved from an extracellular site with approximately 12 amino acids from the plasma membrane. The cleavage product is a 17 kDa molecule that harbours the repetitive CA125 epitope throughout its tandem repeat domains.

CA125, an epitope on the MUC16 molecule, has multiple physiological functions. MUC16 is a large glycoprotein with 22,152 core amino acids and a molecular mass of ~2.5 MDa. MUC16 contains a significant O- and N-linked glycosylated portion with a potential mass of ~20 MDa. It has distinct domains including, the amino-terminal, 60 tandem repeats of 156 amino acids, a transmembrane domain, and a cytoplasmic tail of 32 amino acids rich in tyrosine, threonine, and serine residues used for possible phosphorylation (Figure 1) [58]. The CA125 antigen is normally found in multiple locations within the body. CA125 is present in cervical mucus from healthy women and is likely produced and released from endocervical cells [59]. CA125 is also found in the amniotic fluid and chorionic membrane of the developing fetus in high abundance [60]. It is also expressed in human milk [61], epithelial cells of airways, respiratory glands, and bronchial mucus [62]. Kabawat et al. tested the reactivity of the OC125 monoclonal antibody with different fetal and adult tissues. CA125 was expressed by fetal amniotic, coelomic epithelium, and adult tissues derived from the coelomic and Mullerian epithelia. As a result, CA125 is also expressed in different tissues such as endocervix, endometrium, pleura, pericardium, peritoneum, secretory mammary glands, apocrine sweat glands, intestines, lungs, and kidneys. Additionally, OC125 reacted with adenocarcinomas of the endocervix, endometrium, mesotheliomas, and fallopian tubes. CA125 is present in ovaries’ embryonic development but disappears in the course of development and is then re-expressed in ovarian neoplasms [63,64]. Since CA125 is produced by tissues derived from coelomic epithelium, elevations may be seen in the peritoneal and pleural epithelial and ascites fluids [65]. More importantly, this glycoprotein’s extracellular component is cleaved and shed by ovarian cancer cells and is therefore detectable in body fluids such as serum and peritoneal fluid [66], and amniotic fluid [67].

Multiple groups have tried to target MUC16 therapeutically. In such therapeutics, the tandem repeat domains of MUC16, which are the suspected location for CA125 epitopes, are targeted by antibodies to reduce the chance of ovarian cancer recurrence [68]. Immunotherapeutics such as oregovomab [69] and abagovomab [70] are antibodies against MUC16. 

MUC16 (CA125) plays an active role in ovarian tumorigenesis. MUC16 knockout mice exhibit stalled tumour growth both in vitro and in vivo. In an in vitro experiment, the authors used cell proliferation assays with NIH:OVCAR3 (MUC16 knockdown) and SKOV3 (MUC-16-expressing) cells to investigate the tumour initiation capacity of MUC16 knockout cell lines. Subsequently, their results showed that cell surface MUC16 down-regulation results in the arrest of tumour cell growth in vitro [71]. The in vivo approach included the injection of NIH:OVCAR3 cells into CD-1 nude mice. Tumours derived from the MUC16 knockdown cells were either not detectable or significantly smaller (*p* < 0.0001) [71]. The cytoplasmic tail of MUC16 plays an essential role in tumour growth; in fact, the deletion of the MUC16 C-terminal domain (CTD) inhibits tumour proliferation, cell motility, and invasiveness [71]. The MUC16 gene is among the three most frequently mutated genes in multiple cancer types [72]. MUC16 interacts with numerous other molecules including, Galectin-1 and 3, siglec-9, E and P-selectins, and mesothelin [73]. Rump et al. reported the interactive relationship between CA125 and mesothelin. Mesothelin and CA125 are both present in ovarian adenocarcinomas and are involved in metastases formation by adhesion to the peritoneal mesothelium [74]. Using transwell migration assays, Yuan et al. showed that ovarian cancer cell migration in OVCAR-3 and A2780 cells is significantly enhanced after the addition of CA125. This group also determined that CA125 levels above 82.9 U/mL predict metastasis [75]. In addition to its role in metastasis, MUC16 inhibits Natural Killer (NK) cell-mediated destruction of tumour cells. MUC16 also prevents cancer cell recognition by NK cells; thus, enabling their survival [76]. CA125 is also used as a surface marker for ovarian cancer stem cells. Ovarian cancer stem cells are known as the origin of ovarian cancer recurrence. These stem cells can divide and make new cancerous stem cells, making them valuable targets for therapy [64]. 

MUC16 has shown unsatisfactory results in clinical trials. The ineffectiveness of such antibodies is associated with the site of MUC16 cleavage that is suspected of being near the plasma membrane [77]. MUC16 cleavage takes place in the acidic environment of the Golgi and post-Golgi compartments. The cleavage generates a ~17kDa product [77]. Some groups have focused on targeting the CTD of MUC16, which is retained by the cancerous cells after cleavage. Immunotherapeutic targeting strategies against this domain of MUC16 have shown more promising results [78,79]. Novel therapeutic approaches that involve interference with the cleavage of MUC16 and its interactions with other molecules require further research. 

#### 3.1.2. Factors Influencing Serum CA125 Concentrations

Multiple physiological factors alter normal CA125 serum concentrations. Menopausal status has a profound effect on CA125 levels. The levels of CA125 are substantially higher in healthy premenopausal women [80]. In ovarian cancer patients, CA125 levels are positively correlated with tumour burden and FIGO stage [81]. CA125 is also elevated in multiple benign conditions, leading to many false positives in screening for ovarian cancer. In general, about 20% of ovarian cancers have no CA125 expression. Hence, the expected sensitivity in screening is roughly 80% [34,82]. In a study by Moss et al., 80% of cases with elevated CA125 levels did not have ovarian cancer. Consequently, the high false-positive rate and the modest sensitivity of CA125 in screening contributes to unnecessary surgical procedures and psychological consequences for these women [83]. 

Pauler et al. did a comprehensive analysis of different factors associated with CA125 fluctuations in healthy women. The race was found to be an independent predictor for CA125 levels. African and Asian women tend to have lower CA125 levels compared to Caucasian women. Routine caffeine consumption and smoking were associated with lower CA125 levels. Women with a history of hysterectomy had lower CA125 concentrations, and those with previous cancer diagnoses had higher values. This group also concluded that factors such as woman’s age, age at menarche, age at menopause, and history of ovarian cysts are also related to fluctuations in CA125 levels [84]. Generally, irritation or inflammation to the inner lining of pelvic or abdominal cavities is associated with elevated CA125 levels. For instance, CA125 concentrations are elevated in women diagnosed with endometriosis. CA125 may be able to complement the histological diagnosis of endometriosis because it has sufficient sensitivity and specificity to help with the diagnosis of moderate to severe endometriosis as a rule-in test [85]. CA125 levels are also increased during menstruation. Levels rise during the premenstrual phase and are higher in healthy women with anovulatory cycles [86]. Pregnancy is also associated with minute fluctuations in CA125 concentrations; levels are normal and below the 35 U/mL threshold during the length of gestation. However, the levels start to increase during delivery and continue to rise until 48 h after delivery [87]. In addition, body mass index (BMI) is also positively correlated with CA125 levels [88]. The presence of excess adipose tissue is associated with increased CA125 levels. Consequently, CA125 levels significantly decrease following weight loss by laparoscopic sleeve gastrectomy in obese (mean BMI of 42.71 kg/m^2^) women [89]. CA125 levels naturally decrease as women get older; women between the ages of 45 and 55 show the fastest decline in CA125 levels. Their average serum CA125 levels drop by 30% in 10 years [90].

Fluctuations in CA125 concentrations are also seen in more serious physiological disorders. For instance, CA125 has been proposed as an independent prognostic biomarker for heart failure (HF) patients [91]. Elevations in CA125 concentrations are also observed in 85% of patients with cirrhosis, and detectable levels are seen in ascites fluid from patients with chronic liver disease. Similarly, healthy women with coronary artery disease (CVD) have significantly higher CA125 levels, while others with osteoporosis, osteoarthritis, and hypercholesterolemia have lower CA125 levels than healthy women [92]. Abnormal elevations of CA125 levels are also present in multiple non-ovarian malignancies. For instance, elevated CA125 levels have been known as a prognostic marker in breast and lung cancers [93,94]. For postmenopausal women with high CA125 levels, the presence of lung and breast cancer is recommended to be investigated after the presence of ovarian cancer is excluded [95]. CA125 is also a useful prognostic marker for pancreatic [96], colorectal [97], endometrial [98], and gastric [99] cancers. Adjustment of the CA125 threshold value based on individual characteristics, including a history of benign and malignant diseases, can increase this biomarker’s sensitivity and specificity in screening for ovarian cancer [92]. 

#### 3.1.3. CA125 in Combination with Other Biomarkers

Novel biomarkers could contribute to the accuracy of CA125 and prove their potential as an adjunct marker to CA125. Besides CA125, human epididymis protein 4 (HE4) is the most promising tumour biomarker for ovarian cancer. The two biomarkers have similar sensitivities. However, Ferraro et al. showed that HE4 measurements exhibit a significantly higher specificity than CA-125 (93% vs. 78%) [100]. Multiple other studies have shown improved sensitivity, specificity, and likelihood ratios for HE4 compared to CA125 [101,102]. Andersen et al. developed a symptom index (SI) based on several indicators, including increased abdominal size, pain, early satiety, eating less, and bloating experienced more than 12 times a month. They investigated the combination of SI with HE4 and CA125. A positive test in any two of the three indicators generated a sensitivity of 84% along with a specificity of 98.5%. The addition of imaging studies as a second-line screen would potentially provide a good PPV for ovarian cancer screening [103]. In addition, Urban et al. recommended the replacement of transvaginal ultrasound (TVS) with HE4 as a complementary test to rising CA125 serum levels [104]. In some cases, the combination of HE4 and CA125 can have multiple advantages. For example, HE4 values vary in smokers and contraceptive users, while CA125 values are less affected. On the contrary, HE4 levels are not significantly changed in endometrioma, unlike CA125 [105]. Interestingly, HE4 levels increase with age. Thus, postmenopausal women tend to have higher HE4 levels. The opposite is true for CA125, which exhibits higher levels in premenopausal women with benign conditions. This difference between the expression patterns of HE4 and CA125 may account for the superior performance of HE4 in premenopausal patients, while CA125 performs better in postmenopausal patients [106,107]. 

Multiple biomarker panels involving CA125 have been investigated. Anderson and colleagues evaluated a panel of biomarkers, including HE4, mesothelin, and CA125. The concentrations of these biomarkers started to increase three years before the clinical diagnosis of ovarian cancer [108]. Yurkovetsky and colleagues assessed 96 candidate tumour biomarkers for ovarian cancer screening. Their analysis resulted in a panel of four biomarkers, including CA125, HE4, carcinoembryonic antigen (CEA), and vascular cell adhesion molecule-1 (VCAM-1). This panel had a sensitivity of 86% in early-stage ovarian cancer and 93% in late-stage cancer at a fixed specificity of 98%. About 67% of benign tumours were correctly identified as noncancer. The panel was also specific to ovarian cancer; levels were not elevated in other types of cancer such as breast and lung cancers [109]. In 2019, Russel et al. published their results from a four-protein model capable of diagnosing ovarian cancer 1–2 years before the current methods. The panel included CA125, Vitamin K-dependant protein Z, C-reactive protein (CRP), and Lecithin cholesterol acyltransferase (LCAT), in which CA125 outperformed the other three biomarkers. The panel correctly identified 64% of type II tumours one year before diagnosis and 28% of them two years before clinical diagnosis [110]. In another study, the combination of p53 autoantibodies and CA125 also exhibited promising results as an early detection method for aggressive type II tumours with a sensitivity of 85.7% at a specificity of 100% [56]. Other groups have focused on more complex computational methods such as artificial neural networks (ANN). An ANN combines information from multiple sources and effectively finds sophisticated patterns, using similar mechanisms as the human brain. CA125, CA72-4, CA15-3, and macrophage colony-stimulating factor (M-CSF) values were used as input for ANN. The ANN was found to be superior to CA125 alone for detecting invasive early-stage ovarian cancer. However, the specificity and sensitivity were not sufficient enough to reach a PPV of 10% [111]. 

The search for more accurate ovarian cancer biomarkers is an ongoing process. The majority of the findings suggest that panels of multiple complementary biomarkers can potentially be valuable resources for the early detection of ovarian cancer. Despite the identification of numerous new biomarkers, CA125 is still superior to the majority of novel biomarkers in postmenopausal women, including HE4 [112]. However, practical and cost-effective panels with high clinical accuracy should be the focus of further research. 

#### 3.1.4. Biomarker Based Algorithms Involving CA125

Concerted efforts have been made to improve the sensitivity and specificity of CA125 in screening for ovarian cancer. In addition to biomarker measurements, other variables are useful in estimating the risk for ovarian cancer. Algorithms involving multiple screening modalities, including imaging results, serum biomarker measurements, age, and menopausal status, have been developed to classify patients into different groups based on their risk of having malignant tumours. Algorithms that used CA125 combined with such modalities have been used in multiple settings and are thought to be more valuable than CA125 alone in screening for ovarian cancer. 

One of the first biomarker-based algorithms for ovarian cancer was developed in 1990 to classify women with pelvic masses. The risk of malignancy index (RMI) combined variables such as menopausal status, fixed threshold (≥35 U/mL) CA125 levels, and ultrasound findings into a logistic model [113]. Enakpene et al. investigated RMI’s diagnostic accuracy, an RMI cutoff of 250 lead to a promising sensitivity of 88% and specificity of 74% for preoperative classification of benign vs. malignant adnexal masses. OVA1 is another panel of 5 biomarkers, including CA125, Transthyretin, Apolipoprotein A1, Transferrin, and B2-microglobulin, used to distinguish between the high and low risk of ovarian cancer for patients with pelvic masses. Imaging and menopausal status are also included in this index. This panel has a higher sensitivity than CA125 alone. The next generation of OVA1 is called OVERA; this panel was approved by the Food and Drug Administration (FDA) in 2016. OVERA uses HE4 and follicle-stimulating hormone (FSH) as replacements for B2-microglobulin and Transthyretin used in OVA1. OVA1 and OVERA have high sensitivities (92%, 94%) and low specificities (42%, 65%) [114]. 

Longitudinal screening strategies using biomarker-based algorithms are thought to be superior to screening with single biomarker measurements at fixed thresholds. Jacobs et al. conducted a randomized trial to evaluate ovarian cancer multimodal screening in 22,000 postmenopausal women using sequential CA125 measurements and transvaginal ultrasound. Median survival for cancer patients was higher in the screening arm than the control arm (72.9 months and 41.8 months, *p* = 0.0112). However, no significant difference was observed in the number of deaths from ovarian cancer in the two groups [115]. Using the same dataset, a computer-based cancer risk calculation method using hierarchical changepoint and mixture models was developed. Longitudinal CA125 screening strategy was shown to have superior sensitivity compared to using fixed cutoffs. Serial CA125 levels tend to rise in patients with ovarian cancer, whereas serial biomarker levels tend to stay close to the baseline for those with benign diseases or healthy women. Consequently, the risk of ovarian cancer algorithm (ROCA) was developed using changes in sequential CA125 levels instead of a fixed cutoff value [116,117]. This algorithm classifies patients into different follow-up categories and gives more significance to rapid rises in biomarker levels. 

ROCA was employed in the UK Collaborative Trial of Ovarian Cancer Screening (UKCTOCS) trial to triage women as normal, intermediate, and elevated risk of ovarian cancer. The use of serial CA125 measurements instead of a fixed cutoff of 35 U/mL leads to improved overall performance. Only 4.8 surgeries were needed for each detected case of ovarian cancer. The sensitivity of multimodal screening with serial CA125 measurements and transvaginal ultrasound based on risk was 85.8% at a specificity of 99.8%. Their results reinforced longitudinal screening’s superiority to single cutoff measurements of CA125 [118]. The parametric empirical Bayes (PEB) longitudinal screening algorithm is an alternative method that models the trajectory of biomarker levels in asymptomatic women over time. The information is used to develop positivity thresholds that are specific to each individual. Deviations from the individualized baseline levels of CA125 are associated with a higher risk for ovarian cancer. In general, PEB can detect ovarian cancer earlier than fixed cutoffs. In a study by Drescher et al., sequential CA125 measurements from patients in the Prostate, Lung, Colorectal, and Ovarian Cancer (PLCO) screening trial’s intervention arm were analyzed using PEB. Among the cases that were diagnosed earlier, PEB detected ovarian cancer on average ten months before the fixed cutoff (≥35 U/mL) and at a much lower concentration (20 U/mL) [119]. 

Due to the promise shown by HE4 in multiple studies, this biomarker was included as an adjunct biomarker to CA125 in the risk of malignancy algorithm (ROMA). ROMA accounts for fixed-threshold levels of HE4 and CA125 in addition to menopausal status. ROMA is used to classify patients into high and low risk for ovarian cancer. In the original study of 531 patients, ROMA reached a sensitivity of 92.3% and a specificity of 75% in postmenopausal women [120]. CA125 had a higher false-positive rate (38%) in premenopausal women than HE4 (10%). Benign gynecological conditions, such as endometriosis, are accompanied by elevations in CA125 and RMI values. In such cases, a combination of HE4 and CA125 (ROMA) appears to be a more valuable option [121]. Another group evaluated HE4, CA125, and ROMA in a Turkish population. In their analysis, HE4 and the ROMA index outperformed CA125 with regards to sensitivity (78%, 88%, 63%) at 95% specificity [101]. On the contrary, in a study by Van Gorp et al., ROMA and HE4 did not perform better than CA125 alone. Since CA125 performed better than HE4 in postmenopausal women, the authors concluded that HE4 and ROMA do not improve ovarian cancer diagnosis. The CA125 cutoff value that yielded the highest accuracy was 62.5 U/mL. The authors also concluded that the chance of ROMA and HE4 being an effective biomarker for early-stage screening is low [107]. Chan et al. evaluated CA125, HE4, and ROMA in distinguishing between benign masses and epithelial ovarian cancer in an Asian population. HE4 had lower sensitivity and NPV compared with CA125 but higher specificity and PPV. ROMA showed similar sensitivity as CA125 with slightly improved specificity. ROMA performed better in premenopausal women, which might be due to the lack of HE4 elevations in premenopausal women with benign conditions compared to CA125 [122]. In another study, Moore et al. demonstrated the complementary relationship between HE4 and CA125. ROMA and RMI, which included a combination of ultrasound, CT scan, and MRI, were compared. In their study, ROMA showed more promise than RMI for predicting the risk of early-stage ovarian cancer [123]. Karlsen et al. developed the Copenhagen Index (CPH-I) to diagnose malignant ovarian and fallopian tube masses. In a study of 2665 patients, the sensitivity and specificity of CPH-I were 95% and 78.4%, respectively. The comparison of CPH-I with ROMA and RMI yielded similar results for all three tests. However, CPH-I does not account for imaging and menopausal status. Instead, CPH-I incorporates women’s age in addition to HE4 and CA125 serum levels [124]. The multivariate index assay (MIA) is a blood test assay involving 11 analytes developed to distinguish between ovarian cancer and benign conditions [125]. Later in a prospective trial, MIA combined with clinical assessment performed better than CA125 in both premenopausal and postmenopausal women with early-stage ovarian malignancies (sensitivity of 95.3% vs. 62.8%) [126]. In a comparison between ROMA, MIA, ACOG (American Congress of Obstetricians and Gynecologists) guidelines, CA125 measurements with lowered threshold, and referral for all, the latter two were found to be the most cost-effective options [127]. However, such detection strategies may increase the number of cases per subspecialist, false-positive results, and unnecessary surgeries. 

#### 3.1.5. Modifications of CA125 Cutoff Value

CA125 is more sensitive in postmenopausal women, who account for the majority of ovarian cancer patients. The cutoff value for CA125 is set at 35 U/mL for postmenopausal women, while the same cutoff produces more false positives in premenopausal women. Due to elevations in multiple benign conditions, the American College of Obstetrics and Gynecology recommends a 200 U/mL cutoff value for premenopausal women with pelvic masses based on expert opinion. Premenopausal women with CA125 levels above this cutoff should be referred to a gynecologic oncologist [128]. Approximately 5% of healthy women have CA125 levels above 35 U/mL, while only 0.1% of healthy women have CA125 levels above 100 U/mL [34,129,130]. Moreover, in a study by Van Calster et al., about 75% of ovarian cancer patients have CA125 values above 35 U/mL, while 60% of patients have CA125 levels above 100 U/mL [131]. An increase in the cutoff value of CA125 significantly increases its specificity at the cost of sensitivity [121]. As specificity increases, the number of false positives declines. Simultaneously, the reduction in sensitivity may lead to an increase in false negatives. As a result, many women with early-stage ovarian cancer will be missed by the screening test. The PPV of CA125 depends on the sensitivity, specificity, and prevalence of ovarian cancer. PPV is known as the most useful measure of the effectiveness of a screening test. The PPV of fixed-threshold CA125 measurements in asymptomatic women with a 35 U/mL cutoff is around 1%, corresponding to 1 cancer diagnosed from 100 positive results [34]. Multiple studies have investigated modifications in the CA125 cutoff value in different populations with different ovarian cancer incidences. In a study by Al Musalhi et al., the increased cutoff (≥71 U/mL) for CA125 resulted in a sensitivity of 89% and specificity of 96% compared to the standard cutoff, which yielded a sensitivity of 89% and specificity of 79% in postmenopausal women. An increase in the cutoff for premenopausal women also resulted in improved specificity. This was at the cost of significant reductions in sensitivity for premenopausal women as their normal CA125 values tend to be higher than postmenopausal women [121]. Winarto et al. investigated whether modifications in the standard cutoff values for CA125, HE4, risk of malignancy index (RMI), and risk of malignancy algorithm (ROMA) produce better models for distinguishing malignant and benign tumours. An increase in the cutoff value for CA125 from 35 to 165.2 U/mL resulted in an increase in specificity from 24.6% to 75.4% and a reduction in sensitivity from 96% to 78%. The new cutoff values were associated with higher accuracy for all 4 diagnostic tests [132]. ROMA and HE4 were found to be superior to CA125 alone for detecting ovarian cancer, especially in premenopausal women. The use of all three together can potentially improve testing in premenopausal women suspected of early-stage ovarian cancer. Modifications in the cutoff values for CA125, HE4, and ROMA with respect to menopausal status were investigated for a south Chinese population by Xu and colleagues. They observed increases in the specificity of CA125 with no significant loss of sensitivity when the cutoff was increased to 60 U/mL for premenopausal women [133]. Chang et al. also evaluated cutoff modifications for CA125 and HE4. CA125 thresholds of 127.2 and 325.5 U/mL were obtained at 95% and 98% specificities, respectively. However, like many other studies, the sensitivities declined compared to normal cutoffs [134]. Jacobs et al. investigated the value of multimodal screening (CA125 and pelvic ultrasound) for ovarian and fallopian tube cancers in 22,000 asymptomatic postmenopausal women. Women’s risk of ovarian cancer diagnosis within one year was increased 36-fold if serum CA125 was >30 U/mL and 205-fold if >100 U/mL. The specificity and PPV of CA125 alone were 96.6% and 3.1%, respectively [135]. A shift in focus from screening average-risk asymptomatic women with 35 U/mL cutoff, to the diagnosis of low volume advanced-stage ovarian cancer with higher cutoffs may lead to the detection of more type II ovarian carcinomas in stages IIIa/b where the likelihood of optimal cytoreduction is higher [24,25]. To detect more low-volume advanced-stage ovarian cancers, Spoik et al. evaluated changes in CA125 cutoff from 35 U/mL to 70 U/mL and 100 U/mL, which resulted in specificities of 99% and 99.9%, respectively. This modification also improved the PPV to 5% with a 70 U/mL cutoff and 31% with a 100 U/mL cutoff [34]. 

## 4. Effect of Ovarian Cancer Screening with CA125 on Mortality

Multiple randomized clinical trials have been conducted in the past 20 years to define the value of screening for ovarian cancer in average-risk asymptomatic women. The PLCO trial investigated the effects of early cancer screening on mortality in 10 different centers across the United States. More than 78,000 women (ages 55–74) were randomly assigned to either the screening group, with annual transvaginal ultrasound and CA125 tests (35 U/mL cutoff) or the usual care group. The standard care group did not receive annual screening with CA125 or TVU [112,136]. The extended follow-up data from the PLCO trial indicated no mortality reduction in the study’s screening arm after a median of 15 years of follow-up [137]. A stage shift is cancer diagnosis in earlier stages or earlier within a stage due to screening. The number of advanced-stage cancers was higher in the intervention arm, showing a lack of stage shift. False-positive results increase the number of costly medical procedures at the expense of the patient’s well-being. About 15% of healthy women with false-positive results who underwent surgery experienced surgical complications [136]. Temkin et al. reached some interesting conclusions by analyzing the PLCO data based on the tumour type. Only 15% of type II tumours were screen-detected, while 70% of ovarian cancer patients were suspected of having type II tumours [138]. Therefore, single-threshold CA125 screening with the 35 U/mL cutoff is not the most effective option for the detection of type II tumours, and novel screening techniques need to be developed with a focus on type II tumours. 

The UKCTOCS trial also investigated the impact of early detection on ovarian cancer mortality in 13 centers across England, Wales, and Northern Ireland. More than 202,000 postmenopausal women were randomly designated to one of three groups. The groups included annual multimodal screening (MMS) that incorporates the ROCA algorithm, transvaginal ultrasound, and no screening in a 1:1:2 ratio. MMS had a sensitivity of 89.4% and a specificity of 99.8%, TVS produced comparable results with a specificity of 84.9% and 98.2% specificity. Their primary analysis showed no significant increase in survival. However, later post-hoc analysis showed a moderate increase in survival between years 7–14 of follow-up [31]. One of the differences between the PLCO and this trial is the use of ROCA in the UKCTOCS study. This algorithm incorporates serial changes in CA125 over time rather than a fixed cutoff. Approximately 3% of healthy women with false-positive results experienced surgical complications compared to 15% in the PLCO trial. Xu et al. investigated the predictive value of CA125 velocity in cancer patients and non-cancer patients from the PLCO screening trial. The velocity of CA125 is the rate at which CA125 values increase over a selected period. The velocity of CA125 in ovarian cancer patients was 500 times faster than in healthy women (19.7 U/mL per month vs. 0.035 U/mL per month) [139]. However, in a study by Pinsky et al., the replacement of a single cutoff CA125 screen with ROCA (sequential changes in CA125) in the PLCO trial did not result in a mortality benefit, unlike the UKCTOCS study that showed a modest mortality benefit in years 7–14 of follow-up [140].

Van Nagell et al. evaluated annual ultrasound screening and long-term survival in 37,293 asymptomatic women in the Kentucky ultrasound trial. The 5-year survival rate for women with stage I cancer was 95%, 77.1% for those with stage II cancer, and 76.2% for stage III tumours. Those with abnormal ultrasound results received CA125 testing and, eventually, surgery if CA125 levels were elevated. The survival rates were substantially higher for women in the screening group, and more early-stage cancers were diagnosed [141]. Gupta et al. detailed the presence of a “healthy volunteer effect” in the Kentucky ultrasound study. Volunteers in clinical trials usually have better survival outcomes compared to the general population due to multiple reasons. Volunteers tend to be from higher income and educational backgrounds. Healthy volunteers who report themselves as unhealthy are usually excluded, and the exclusion of volunteers with poor health is sometimes seen in such studies. However, this bias is seen in multiple clinical trials, including the PLCO trial and is hard to correct for [142,143]. Another randomized trial involving 83,487 asymptomatic postmenopausal women was completed in Japan. The intervention group received an annual pelvic ultrasound and CA125 tests. Despite the high number of false positives, the presence of a stage shift was evident upon analysis. After a mean of 9.2 years of follow up, the difference between the number of screen-detected stage I cancers in the screening and control groups was not statistically significant. Mortality results from this trial are yet to be published [144]. 

BRCA1/2 mutation carriers are at a higher risk of developing ovarian cancer. Routine screening in BRCA mutation carriers with transvaginal ultrasound and CA125 is currently not recommended [145]. Van der Velde et al. investigated 241 women with BRCA1/2 mutations to evaluate ovarian cancer screening in a high-risk population. Their screening method included annual transvaginal ultrasound (TVS), pelvic examination, and CA125. No early-stage ovarian cancers were detected. The authors concluded that annual screening for BRCA1/2 mutation carriers is not sufficient [146]. 

Aggressive cancers progress more quickly beyond the early stages of ovarian cancer. A screening model that incorporates aggressive and indolent phenotypes based on histopathologic criteria determined that ovarian cancer stays in stage I for an average of 8.1 months in the aggressive phenotype and 23.7 months in the indolent phenotype. The difference between indolent and aggressive subtypes of ovarian cancer has implications on the current screening methods and potential improvement in outcome. The implications include a short window for detecting aggressive cancers; patients with aggressive cancers must be diagnosed as soon as possible while optimal cytoreduction is achievable. Using this model, the estimated mortality reduction by annual screening in average-risk women is limited [147]. The American Cancer Society does not recommend screening for ovarian cancer in asymptomatic women without a family history or genetic predispositions. Those with genetic risk factors are eligible for screening with TVS and CA125, although the benefits are still controversial [146,148]. Despite the common perception, the value of the early diagnosis of ovarian cancer is also surrounded by controversy. Early detection of ovarian cancer (stages I and II) has failed to provide a considerable reduction in mortality. An early-stage ovarian cancer diagnosis is synonymous with the detection of type I tumours associated with a more favourable prognosis and account for a small proportion of ovarian cancer mortality. The volume of residual disease after cytoreductive surgery is the most powerful prognostic factor for ovarian cancer patients. Therefore, the focus of screening should be shifted towards the diagnosis of low volume disease irrespective of stage [149]. New cost-effective and accurate screening techniques that can detect type II ovarian carcinomas early in their evolution may substantially impact survival for ovarian cancer patients.

## 5. Recurrent Ovarian Cancer and CA125

Survival rates for most cancers have been increasing for the past three decades. The increased survival is mostly due to advances in screening, surgical procedures, and treatment strategies [150,151]. Nonetheless, ovarian cancer recurs in 25% of early-stage and more than 80% of advanced-stage patients [150]. Advances in the treatment (i.e., chemotherapy) of ovarian cancer appear to have minimal effect on mortality, as the 12-year survival rates have improved modestly since the introduction of chemotherapy [152]. Recurrent ovarian cancer is linked to significant reductions in survival and is generally characterized as incurable [23]. The 5-year and 12-year survival rates for recurrent ovarian cancer are less than 30% and 5%, respectively [23,153]. Screening for ovarian cancer recurrence involves physical examination, imaging, and serum CA125 monitoring. About 80–90% of all recurrences can be diagnosed by physical exam and serum CA125 levels [154,155]. Additionally, various imaging techniques can be used to accompany tumour biomarkers and detect the remaining recurrences of ovarian cancer. In a study by Salani et al., patients with zero residual disease after secondary cytoreductive surgery survived longer than those with macroscopic residual disease (50 vs. 7.2 months, *p* < 0.01) [156]. As recurrence prevention is equivalent to the prevention of death, effective screening techniques that include CA125 may help with the rapid diagnosis of recurrence, leading to a higher likelihood of optimal secondary cytoreduction. 

In addition to its role as a diagnostic marker for recurrent ovarian cancer, CA125 is also used as an indicator of response to treatment. Before adopting CA125 as a marker for ovarian cancer progression, physical examination and imaging modalities were used as the primary diagnostic tools. However, the sensitivity of such techniques was only sufficient in patients with large tumours in the pelvic area [42]. CA125 has been the most clinically useful biomarker for predicting and managing disease recurrence in ovarian cancer patients. Rising and falling values of CA125 are predictors of disease progression and regression in 90% of cases. Furthermore, a rise to more than double the normal CA125 threshold concentration of 35 U/mL is a valid predictor of tumour relapse after the first-line chemotherapy [157,158,159]. CA125 can detect ovarian cancer recurrence with 62–94% sensitivity and 91–100% specificity [150]. Patients with clinical remission that have three progressively rising CA125 measurements within the normal range (<35 U/mL) have a substantially higher risk of recurrence [160]. 

### 5.1. CA125: Early Detection of Relapse

Elevations in CA125 has been shown to detect recurrent cancer 2–5 months before clinical diagnosis [161]. In a study by Rustin et al., the specificity and sensitivity of CA125 for predicting recurrent ovarian cancer were 91.3% and 85.9 %, respectively. When the doubling concentration of CA125 outside the 35 U/mL cutoff was confirmed with a second CA125 test, the false positive rate was reduced to <2%. A median lead time of 63 days was observed for patients diagnosed with serum CA125 levels as opposed to clinical diagnosis [157]. A CA125 level of 1.68 times the nadir value is also an indicator of recurrent cancer. Asymptomatic patients who have undergone secondary cytoreductive surgery based on CA125 levels have more prolonged progression-free survival (PFS) than patients with clinical relapse [162].

Rustin et al. also investigated early treatment based on CA125 elevations versus delayed treatment based on clinical symptoms. Five hundred twenty-nine patients were randomly distributed into early and delayed treatment groups. Patients in the early-treatment arm started second-line and third-line chemotherapy 4.8 and 4.6 months earlier, respectively. After a median of 56.9 months of follow-up, the authors found no sign of survival advantage with early treatment based on an elevated CA125. The patients who were treated earlier showed deteriorations in quality of life, likely due to chemotherapy [163]. This study undermined the value of follow-ups with CA125 in clinical practice. The authors recommended that patients should be consulted and make an informed decision based on the available evidence. Several limitations of this study have been mentioned in the literature. Rustin et al. used a doubling of the CA125 levels from the upper limit as the indicator for cancer progression. However, increases in CA125 concentrations below the cutoff value have been linked to disease progression [161,164]. Some ovarian cancer patients show complete serological response to treatment; their CA125 levels return to normal (≤35 U/mL) and might only increase in small increments. For patients with complete response to therapy, a rise of 5 U/mL or 10 U/mL from the nadir CA125 level has been correlated with recurrence [161]. Additionally, delays in treating the patients in the early arm and a lack of carboplatin and taxane combination in chemotherapy might account for the lack of survival in the early-treatment group [165]. 

Women with recurrent ovarian cancer experience a low overall quality of life due to poor physical and emotional conditions [153]. Hopkins et al. developed a decision model to describe the use of CA125 in the follow-up of women with advanced ovarian cancer from a societal perspective. They concluded that most patients favour test results to be used in the course of their treatment but recommended that CA125 measurements should be used only after an explanation and interpretation from the physician on the preference-sensitive nature of CA125 [166]. In a study by Tanner et al., more than 80% of women with recurrent ovarian cancer were diagnosed by CA125 and/or imaging in an asymptomatic period. These women survived on average 21 months (*p* = 0.004) more than those with clinical diagnoses based on symptoms. Asymptomatic patients had more optimal secondary cytoreductive surgeries and better chemotherapy response than those with a clinical diagnosis [167]. This study showed the value of the early diagnosis of recurrence in improving patients’ outcomes. 

### 5.2. CA125: Prognostic Value

CA125 concentrations below 35 U/mL after first-line chemotherapy in late-stage ovarian cancer patients represent improved overall survival (OS) and disease-free survival (DFS) [158,167]. After platinum-based chemotherapy, a concentration change was shown to be an independent prognostic factor in stage III and IV patients. Specifically, a reduction of CA125 to less than 50% of pre-treatment value after two cycles of platinum-based chemotherapy is associated with improved survival [168].

CA125 nadir concentration and half-life are also powerful independent prognostic indicators. In a French multicenter study by Riedinger et al., a nadir CA125 values below 20 kU/L were associated with more prolonged overall survival and disease-free survival (*p* < 0.0001). The time elapsed from the start of treatment to reach the nadir value was also an important prognostic indicator. Patients who reached their nadir CA125 levels in less than 72 days had better DFS and OS (*p* < 0.0001). Similarly, low CA125 half-life (<14 days) and low CA125 pre-treatment values (<230 kU/L) were also associated with improved DFS and OS (*p* < 0.0001) [169]. Another group reported that a half-life of <25 days translated into a 3.585-fold increased chance of achieving a complete response to treatment (*p* < 0.0001) [170]. Other important prognostic factors for ovarian cancer recurrence include the FIGO stage, presence of ascites, and size of residual disease after surgery [167,170]. The interval between CA125 elevation and secondary surgery is crucial for successful treatment. Fleming et al. proposed a shorter time interval between CA125 elevation (two times the nadir value) and subsequent secondary cytoreductive surgery, leading to optimal resection at surgery (≤0.5 cm residual disease). They concluded that surgery one week after initial CA125 elevation can lead to a 3% increase in the chance of suboptimal resection and may result in a shorter DFS and OS [171]. Other tumour markers have been compared to CA125 as potential prognostic factors in predicting ovarian cancer recurrence. Cancer-associated serum antigen (CASA), an epitope on a mucinous protein from the MUC1 gene, is one of those markers. Gronlund et al. reported CASA as an independent prognostic indicator for survival, while pre-treatment CA125 concentrations were not a sufficient independent prognostic factor at different cutoffs [172]. However, in a meta-analysis by Gu et al., CA125 was the most specific (93%) prognostic indicator for ovarian cancer recurrence. Various imaging techniques can increase the accuracy of CA125. PET/CT was the most sensitive and was recommended as a supplement to CA125 [173]. 

## 6. Future Directions: Ovarian Cancer Screening

The current ovarian cancer screening techniques face many challenges. Biomarkers and imaging modalities are yet to provide a cost-effective and reliable screening strategy that leads to improvements in outcome. The new ovarian cancer pathogenesis model divides the known ovarian cancer subtypes into types I and II tumours. Diagnosis of ovarian cancer in stages I and II have been the focus of numerous screening trials. However, by targeting such tumours, we are more likely to diagnose “good prognosis” type I tumours [149]. Type II ovarian cancer is the most aggressive subtype, which progresses very quickly (under one year) to stage III. Therefore, the diagnosis of low volume (stage IIIa/b) type II tumours should be the primary goal of screening.

**Figure 2:**
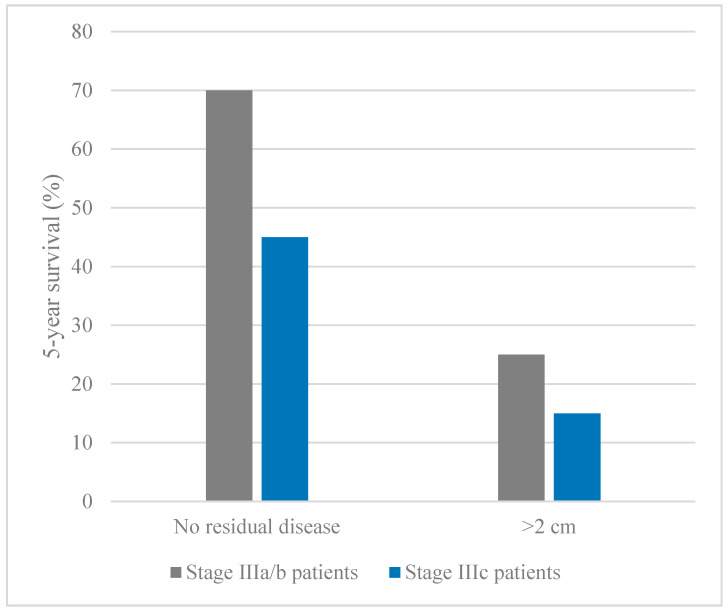
5-year survival in stage IIIa/b and IIIc patients with optimal and suboptimal resection at primary cytoreductive surgery [26,174].

Additionally, type II tumours are usually diagnosed at stages III and IV, where they quickly progress beyond stages IIIa/b to stages IIIc and IV. The majority of HGSCs (84%) are diagnosed at stage IIIc [174]. Stage IIIa/b patients have microscopic or small but visible (<2 cm) abdominal metastases or retroperitoneal lymph node metastases [175]. Such patients are more likely to undergo optimal cytoreduction and achieve the status of no residual disease which is associated with higher 5-year survival (Figure 2). Optimal cytoreduction is less likely in stage IIIc patients who have bulky upper abdominal disease [24,25]. Therefore, due to the rapid progression of type II tumours, patients with stage IIIa/b tumours should be diagnosed as fast as possible before progressing to stage IIIc. 

**Figure 3:**
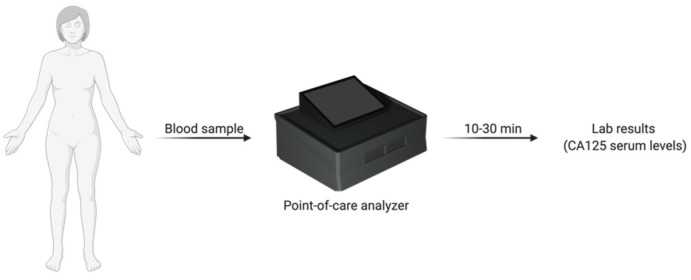
Point-of-care biomarker analyzer for primary care facilities.

Easily accessed and cost-effective point-of-care screening devices could be employed in primary care facilities for enhanced ovarian cancer screening. Point-of-care analyzers are portable and user-friendly systems that allow for the quantification of biomarkers within minutes. Point-of-care systems could help with the diagnosis of more type II tumours in surgically manageable and low volume stages within the primary care facility. Women with an initial indication of pelvic mass or ovarian cancer-related symptoms and also women with nonspecific symptoms can undergo rapid CA125 testing (Figure 3). This method has the potential for the detection of more tumours at stage IIIa/b, which increases the chance of optimal cytoreduction at primary debulking surgery. In addition, since longitudinal screening is superior to single threshold CA125 measurements for ovarian cancer screening in average-risk women, easily accessed point-of-care CA125 measurements can be used to facilitate more effective longitudinal screening [176]. 

Multiple groups have investigated different methods for reducing the false-positive rate associated with CA125 in screening. These efforts include the personalization of cutoff values based on menopausal status and oral-contraceptive usage [80]. Point-of-care devices also allow for the personalization of biomarker cutoff values. More importantly, the cutoff value of CA125 can be modified with regards to the CA125 expression patterns of type II tumours. The majority of type II tumours, which mostly become symptomatic at stage III, have CA125 concentrations above 100 U/mL. Whereas only a small percentage of healthy women have CA125 levels above this value. [34,49,129,130,131]. By increasing the cutoff value of point-of-care CA125 tests to 100 U/mL, we can potentially increase the diagnosis of type II ovarian cancers in stages IIIa/b. This modification has the potential to reduce the false-positive rate associated with ovarian cancer screening. Reductions in the false-positive rate may be accompanied by mild reductions in sensitivity and could lead to missing some early-stage (stages I/II) patients. However, the rapid diagnosis of stage IIIa/b patients should be of greater priority. The capability of diagnosing such patients in a primary care facility provides enough time for efficient management and, potentially, a greater chance of optimal cytoreduction and survival.

### Chip-Based and Cartridge-Based Biosensors

The ideal screening strategy involves cost-effective and easy to use methods that can be employed in a point-of-care setting. The traditional assays used for the quantification of CA125 include the use of radiolabeled monoclonal antibodies in a sandwich-type assay. After CA125 binds the antibodies, the corresponding radioactivity is synonymous with the quantity of CA125 available in the sample. This type of assay is not the most cost-effective technique, requires automated technology, and is time-consuming. 

Microfluidic biosensors have gained considerable attention as point-of-care devices in the past decade. Lab-on-chip biosensors can quantify biomarker concentrations using different types of electrochemical assays. The combination of biosensors and microfluidics has the potential for cost-effective point-of-care detection of biomarkers, including CA125, leading to earlier diagnosis of advanced-stage ovarian cancer. Shang et al. developed a microfluidic chip-based sandwich assay with lectins and antibodies. The chip is imaged by fluorescent microscopy and then analyzed for CA125 quantification. The LOD of CA125 detection was close to commercial CA125 ELISA kits [177]. Zhao et al. developed an ExoSearch chip (microfluidic continuous flow platform) for multiplexed exosome detection. Exosomes are vesicles that carry several proteins, DNA, RNA, and other soluble particles. The chip was used to detect CA125, EpCAM, and CD24 in the plasma of ovarian cancer patients. The ExoSearch method includes an in situ multiplexed exosome immunoassay that can rapidly identify exosomes and their components. This chip requires small amounts of plasma (20 μL) and detects the presence of the three biomarkers in 40 min. Using this method, women with elevated CA125 and possibly other biomarkers such as HE4 can be identified [178].

Similarly, the microfluidic biosensor developed by Nunna et al. uses changes in capacitance as CA125 binds its antibody linked to gold nanoparticles. This technique also allows for the employment of multiplex assays involving panels of biomarkers, leading to reduced false positives and false negatives in screening for ovarian cancer [179]. Another group developed electronic biosensors with interdigitate electrodes (IDEs) to detect ovarian cancer biomarkers CA125, CEA, and HE4 in serum samples. In this technique, once the biosensor detects the biomarkers, the generated signal is measured by electrochemical impedance spectroscopy (EIS). Furthermore, aptamers are used instead of antibodies to enhance binding to antigens. An aptamer is a single strand of DNA (ssDNA) with the ability to bind proteins. Multiple aptamers have been developed to improve binding with CA125 [180,181]. Aptamers are thought to be superior to antibodies. Their production is more cost-effective; they are modifiable, easier to include in biosensor devices, and are more stable. Even though nucleases readily destroy aptamers, certain modifications to their ends can prevent reactions with nucleases [181].

A 3D microfluidic origami electrochemiluminescence chip was also employed for the detection of CA125 in serum. Multiple methods in a short time can complete the fabrication process for this chip. In the original study, wax-patterned paper fluidic substrates and screen-printing electrodes were used to fabricate the device. This technique involved the use of two CA125 antibodies with different binding sites in a sandwich-type immunosensor. This immunosensor showed increased sensitivity and a low minimum detection limit of 0.00074 U/mL [182]. Another chip was developed by Shadfan et al. and is capable of quantifying concentrations of CA125, HE4, CA72-4, and MMP-7. Similar to other nano-chips, it provides a robust and rapid (43 min) test that can be used in a POC setting. This chip’s sensitivity and specificity in the diagnosis of cancer samples were 68.7% and 80%, respectively [183]. Microfluidic biosensors usually use urine or blood as samples. Recently, Williams et al. developed an implantable device that can be placed near the cancer site (ovaries, fallopian tubes, peritoneal cavity) where biomarker concentrations are greater compared to serum. The device is comprised of carbon nanotube complexes that can detect HE4 on the nanomolar scale. The carbon nanotubes can transmit near-infrared (NIR) bandgap photoluminescence between 800 and 1600 nm. The fluorescence can be visualized endoscopically or from the body surface. The authors investigated only one biomarker (HE4) while there is the possibility for multiplex detection of a panel of biomarkers for improved accuracy. High-risk women and patients with primary ovarian cancer are the primary candidates for this technology, allowing for earlier detection of ovarian cancer recurrence [184]. An interesting approach was taken by Hosu et al. in the development of a smartphone-based CA125 immunosensor. In a sandwich-type assay, the primary antibodies are fixed onto a 3D nitrocellulose membrane, while the secondary antibodies are labelled with gold nanoparticles (AuNPs). After a silver enhancement reaction, the gold-silver nanoparticles result in different grey colour spots, quantified by a smartphone camera. This method can quantify serum CA125 concentrations in the range of 30–1000 U/mL [185].

In addition to microfluidic approaches, multiple cartridge-based POC immunoassay systems have been developed. In such methods, the cartridge has all the required reagents for analyzing the specimen. After insertion of the cartridge, the analyzer automates all the required steps and reads the emission results [186]. For instance, the QL analyzer developed by Cardiogenics is a cartridge-based POC system with quantitative chemiluminescence immunoassay capabilities. This analyzer contains a self-metering cartridge and reports the test results within 15 min [187]. 

Some limitations and challenges involved with such biosensors include a lack of automation leading to less tested samples (Table 3). Additionally, the aptamer or antibody needs to be in the correct orientation upon binding with antigen; otherwise, the device lacks optimal binding. In addition, since blood serum has considerable surface fouling, the device must be optimized for such samples [188]. Microfluidic-based and cartridge-based immunoassays can provide cost-effective and rapid testing in a POC setting, leading to considerable advances in screening for ovarian cancer (Table 3). They also allow for longitudinal biomarker monitoring, which is proven to be more useful than fixed cutoffs. This method can particularly be useful for high-risk women with BRCA1/2 mutations or a family history of breast or ovarian cancers who need more frequent serum biomarkers measurements. This technique’s accessibility and robustness can also be useful in developing countries with limited infrastructure for effective laboratory-based screening of both average-risk and high-risk women. 

## 7. Conclusions

Ovarian cancer is a heterogeneous disease with variable prognoses in different subtypes. Patients with type I tumours are typically diagnosed in the early stages and have a favourable long-term prognosis. Conversely, patients with aggressive type II tumours are diagnosed at advanced stages with a poor survival outcome. CA125 is a high molecular weight glycoprotein that has served as the main ovarian cancer biomarker for almost four decades. CA125 has played an important role in the screening, treatment, and follow-up phases of ovarian cancer management. Early detection of ovarian cancer can have a considerable impact on improving survival rates for both types of tumours. Early diagnosis of ovarian cancer at stages I and II through screening with CA125 has shown little promise. However, the recent paradigm shift towards low volume type II ovarian cancer diagnosis may lead to more exciting results. The diagnosis of type II tumours early in their evolution requires the availability of easily accessed and cost-effective screening techniques. POC assays with increased cutoff values and longitudinal CA125 monitoring capabilities could be effective options compared to screening average-risk women with single threshold CA125 measurements. POC CA125 measurements may also have the power to detect advanced stage type II ovarian cancer in earlier substages (stage IIIa/b) where optimal cytoreduction is more likely. In addition, the combination of other biomarkers with CA125 can increase the sensitivity of this biomarker in diverse populations. Therefore, we believe that the current CA125-based screening techniques will have to be reconsidered if we are to make significant reductions in ovarian cancer mortality.

## Figures and Tables

**Table 1:** Type I versus type II ovarian carcinomas.

	Type I	Type II
Subtypes	Endometrioid, clear cell, low-grade serous carcinomas (LGSC), mucinous carcinomas, seromucous carcinomas, malignant Brenner tumours	High-grade serous carcinoma (HGSC), carcinosarcoma, undifferentiated carcinoma
Genetic stability	Genetically stable	Genetically unstable
Diagnosis	Early-stage	Advanced-stage
Early detection	Frequent	Infrequent
Progression	Slow	Rapid
TP53 mutations	Infrequent	Frequent
Germline BRCA mutations	Infrequent	Frequent
Ki 67 proliferative index	10–15%	50–75%
Median CA125 levels	53–413 U/mL	395–1340 U/mL

**Table 2:** Median CA125 levels (U/mL) in women with benign adnexal masses, type I, and type II ovarian tumours.

Study	*n*	Benign	Type I	Type II
Alcázer et al. [15] (2013)	244	NA *	78.9	490
Leandersson et al. [50] (2016) †	350	54	413	1340
Kristjansdottir et al. [51] (2013)	373	16	53	395
Gąsiorowska et al. [53] (2015)	206	25	45	936
Yanaranop et al. [54] (2018)	499	35.8	155.8	690.7
Liu et al. [55] (2017) †	65	NA *	141.1	299.9
Fujiwara et al. [56] (2015)	225	21.9	61.2	567.2

† Values represented as mean. * All women had confirmed epithelial ovarian cancers.

**Table 3:** Advantages and disadvantages of point-of-care screening.

Advantages	Disadvantages
Easily accessible at the physician’s office and patient’s bedside	Analysis of a few samples
Portable	Antibodies, aptamers, or lectins need to be in correct orientations
Low cost (equipment and personnel)	Quality control and calibration
Rapid results	Error management and interassay variations
Multiplex testing for several biomarkers	Analysis time of up to 3 h for some microfluidic devices
Minimal sample requirement	Distribution to primary care facilities
Minimal sample processing	
